# Insights into the evolutionary history and widespread occurrence of antheridiogen systems in ferns

**DOI:** 10.1111/nph.16836

**Published:** 2020-08-25

**Authors:** Ondřej Hornych, Weston L. Testo, Emily B. Sessa, James E. Watkins, Courtney E. Campany, Jarmila Pittermann, Libor Ekrt

**Affiliations:** ^1^ Department of Botany Faculty of Science University of South Bohemia Branišovská 1760 České Budějovice CZ 37005 Czech Republic; ^2^ Department of Biology University of Florida Box 118525 Gainesville FL 32611 USA; ^3^ Department of Biology Colgate University 13 Oak Drive Hamilton Hamilton NY 13346 USA; ^4^ Department of Biology Shepherd University PO Box 5000 Shepherdstown WV 25443 USA; ^5^ Department of Ecology and Evolutionary Biology University of California Santa Cruz CA 95060 USA

**Keywords:** antheridiogen, apomixis, ferns, gametophyte, germination, mating, polyploidy, sex expression

## Abstract

- Sex expression of homosporous ferns is controlled by multiple factors, one being the antheridiogen system. Antheridiogens are pheromones released by sexually mature female fern gametophytes, turning nearby asexual gametophytes precociously male. Nevertheless, not all species respond. It is still unknown how many fern species use antheridiogens, how the antheridiogen system evolved, and whether it is affected by polyploidy and/or apomixis.
- We tested the response of 68 fern species to antheridiogens in cultivation. These results were combined with a comprehensive review of literature to form the largest dataset of antheridiogen interactions to date. Analyzed species also were coded as apomictic or sexual and diploid or polyploid.
- Our final dataset contains a total of 498 interactions involving 208 species (*c*. 2% of all ferns). About 65% of studied species respond to antheridiogen. Multiple antheridiogen types were delimited and their evolution is discussed. Antheridiogen responsiveness was not significantly affected by apomixis or polyploidy.
- Antheridiogens are widely used by ferns to direct sex expression. The antheridiogen system likely evolved multiple times and provides homosporous ferns with the benefits often associated with heterospory, such as increased rates of outcrossing. Despite expectations, antheridiogens may be beneficial to polyploids and apomicts.

## Introduction

Homospory (the production of a single spore type at meiosis) is presumed to be the ancestral state in land plants, yet the majority of extant species are heterosporous (producing two types of spores, typically male and female, at meiosis) and heterospory evolved a minimum of 11 times (Bateman & DiMichele, 1994). Heterospory promotes genetic diversity by limiting inbreeding (Qiu *et al*., 2012). In contrast, gametophytes of homosporous plants can be bisexual and are theoretically capable of gametophytic selfing, that is the fusion of two gametes originating from a single gametophyte via mitosis (Haufler *et al*., 2016). As the gametophyte grows from a spore originating via a single meiotic event, the sporophyte arising from gametophytic selfing is completely homozygous (Klekowski & Lloyd, 1968). Nevertheless, contemporary homosporous lineages maintain their genetic diversity by mechanisms that reduce the rate of gametophytic selfing. Some bryophyte gametophytes have their sex determined via sex chromosomes (Renner *et al*., 2017), whereas fern gametophytes often use a dynamic system controlling sex expression via pheromones called antheridiogens (Schneller, 2008).

Walter Döpp first discovered antheridiogen (hereafter abbreviated AG) in 1950, originally named ‘A‐substanz’. During his experiments with gametophyte cultivation, Döpp noted that reusing agar media previously used to cultivate *Pteridium aquilinum* gametophytes caused precocious formation of antheridia in young gametophytes of *Dryopteris filix‐mas* (Döpp, 1950). This effect was confirmed by Näf in 1956 and attributed to a pheromone exuded by older gametophytes that was later named antheridiogen (Näf *et al*., 1975). Since the discovery of AG, evidence of the utilization of the pheromone has been documented in some (but not all) fern species that have been tested across phylogenetically disparate lineages (Schneller, 2008).

Available evidence suggests that AG production and response varies considerably among fern taxa and that the system involves complex inter‐ and intraspecific interactions. This has been evident since Döpp's initial discovery of AG as his report involved taxa belonging to two different families. Later studies revealed that AGs often have a gibberellin‐like structure (Yamane, 1998) and indicated that various types of AGs occur across the fern clade (Schneller *et al*., 1990). Generally, AGs have been classified either by the species producing them (e.g. A_An_ for AG released from *Anemia phyllitidis*) or in broad groups/types according to the taxa that they affect. Three main types of AGs typically are recognized under the second classification scheme (Schneller *et al*., 1990). First, A or A_Pt_ type is used widely by many species throughout the order Polypodiales, notably by *P. aquilinum* and *Onoclea sensibilis*. Second, B or A_An_ type is used only within the order Schizaeales, notably by *Anemia* and *Lygodium*. Interestingly, gibberellins known from seed plants can evoke the same response as the A_An_ type (Voeller, 1964; Weinberg & Voeller, 1969). Finally, C or A_Ce_ type is used exclusively by the genus *Ceratopteris*. Several other types have been described by a limited number of studies, for example in *Asplenium ruta‐muraria* by Schneller & Hess (1995).

Although several distinct types of AGs were described, the primary function of all AGs is the stimulation of precocious formation of antheridia. When a gametophyte of an AG‐responsive species grows in the absence of this pheromone, it first develops archegonia (i.e. becomes female; Döpp, 1950). However, right before the gametophyte reaches the archegoniate phase, it begins exuding AGs into its environment (Näf *et al*., 1975). At the same time, the gametophyte loses the ability to respond to AGs (Näf, 1958). Younger or slow‐growing asexual gametophytes in the immediate surroundings of the first gametophyte respond to the AGs by halting growth and forming antheridia (i.e. becoming male). The population ends up composed of a few larger female gametophytes and many smaller male gametophytes (Tryon & Vitale, 1977). As fern sperm are flagellated and need to swim through water to reach archegonia, a greater abundance of sperm due to the AG system may help overcome the limitations of dry environments (Schneller & Hess, 1995). Likewise, AG leads to a greater number of unisexual gametophytes, therefore limiting self‐fertilization and facilitating outcrossing, the exchange of gametes between gametophytes, and therefore maintaining heterozygosity in fern populations (Schedlbauer & Klekowski, 1972). Through the AG system, homosporous ferns gain these advantages, which are usually afforded to heterosporous plants because of their pre‐determined sexes and consequent inability to undergo the extreme form of selfing found in homosporous plants (Bateman & DiMichele, 1994). Additionally, larger gametophytes may be able to pheromonally suppress the ability of smaller gametophytes to bear sporophytes, thus reducing competition. However, smaller gametophytes may use the system to contribute to the next generation despite being unable to form archegonia or support young sporophytes owing to unfavorable conditions (Willson, 1981).

Generally, fern spores require light to germinate, but AG was found to replace the need for light in spores cultivated in complete darkness (Raghavan, 1989). In nature, spores buried under a thin layer of soil or detritus affected by AG may form tiny gametophytes and reach the surface or use their limited resources to form a small number of antheridia, skipping the archegonial phase (Schneller, 1988). The sperm from those gametophytes then can reach female gametophytes aboveground (Haufler & Welling, 1994). Therefore, AG enables the mobilization of the genetic and sperm‐producing potential of spores buried underground. The concentrations of AG needed to stimulate the precocious formation of antheridia and germination in darkness may differ (Schraudolf, 1962; Weinberg & Voeller, 1969; Endo *et al*., 1972). If the two effects, germination in darkness and precocious antheridium formation, are tightly correlated, dark germination could be used to test the response to AGs in multiple species, as was done by Weinberg & Voeller (1969). However, most authors comparing the two effects of AGs within one study have only focused on a few species (Yamane *et al*., 1987; Chiou & Farrar, 1997; Chiou *et al*., 1998) and a thorough review is necessary.

In theory, some fern species may gain very little but lose a lot by responding to AGs. For example, neopolyploid species (*sensu* Vida, 1976; herein after referred to as polyploid), having more than two sets of chromosomes and therefore the potential to ‘buffer’ against the deleterious effects of gametophytic selfing, may reproduce by self‐fertilization and still retain genetic variation (Klekowski & Baker, 1966; Hickok, 1978). So, polyploids should tend to self‐fertilize more than diploids (Masuyama, 1979; Soltis & Soltis, 2000; Sessa *et al*., 2016). As the AG system limits self‐fertilization, polyploid species may be more likely to stop using the pheromone, potentially allowing all polyploid gametophytes to bear sporophytes and thus avoid any negative adverse effects. However, no comparison of AG response between diploids and polyploids has been conducted until now. A more extreme case of AGs as a potential liability exists in apomictic ferns. Apomictic gametophytes form sporophytes apogamously from a somatic cell, without the need for fertilization. This renders any extra sperm present in a population as a response to AGs presumably useless. Nevertheless, the ability to suppress surrounding gametophytes may be potentially advantageous from the standpoint of reducing competition. The limited number of tested apomicts were found either to respond to AGs (*Bommeria pedata*, Haufler & Gastony, 1978; *Dryopteris affinis*, Schneller, 1981) or ignore AGs (*Cyrtomium* spp., Yatskievych, 1993). However, a thorough study of AG response in apomictic ferns has not yet been conducted.

Despite the apparently widespread occurrence of AG systems in ferns and their potentially large evolutionary significance via their effects on population structure and mating behavior, our understanding of their evolution and distribution across the fern phylogeny remains limited. Several authors have put together lists of all ferns tested for AG response (Näf *et al*., 1975; Raghavan, 1989; Schneller, 2008) but we are unaware of any attempt to evaluate AG systems in a broader evolutionary context (with the exception of Greer *et al*.*,* 2009) which incorporated only the handful of species responding to gibberellins). To determine how widespread the involvement of AGs is among ferns, we combined results of our cultivation experiments with a meta‐analysis of all published results of similar assays available to us, including 208 species in total. Using this large dataset, we address the following questions: How many fern species have been tested for AG response and how many of those respond? How many different types of AGs appear to exist and what is their evolutionary history? How tightly are the two effects of antheridium induction and germination in darkness correlated? How are AG production and response correlated with ploidy level and reproductive mode?

## Materials and Methods

### Cultivation

Frond material with mature sporangia of 69 fern species from 19 families was collected from wild or cultivated plants (Supporting Information Table S1) from tropical and temperate regions. Fronds were allowed to air‐dry in paper envelopes to facilitate spore release.

Spores were sown on 1% agar supplemented with standard Bold's medium (Bold, 1957) modified with Nitsch's micronutrients (Nitsch, 1951) in 100 × 25 mm Petri plates. Surface sterilization of spores was not performed, and no fungal contamination was observed within the test period. For all cultivation experiments (with or without mature gametophytes), spores were sown at an approximate density of 25 spores per 100 × 25 mm Petri plate (ThermoFisher Scientific, Waltham, MA, USA) by dispersing them through pinholes from glassine envelopes. Cultures were kept at 25°C and exposed to a 12 h : 12 h, light : dark photoperiod achieved with fluorescent grow bulbs (65 μmol m^−2^ s^−1^) in growth chambers (BioChambers, Winnipeg, MB, Canada and Percival Scientific, Perry, IA, USA).

**Fig. 1:**
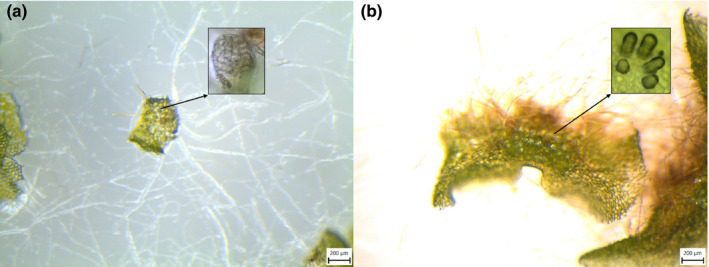
Gametophytes of *Asplenium ruta‐muraria* with gametangia at magnification used for sex determination in this study with details at higher magnification presented in rectangles: (a) male gametophyte bearing antheridia and (b) female gametophyte bearing archegonia.

In order to test the potential influence of antheridiogen (AG), spores of each species were sown in the presence of either a single conspecific or a single *Pteridium aquilinum* archegoniate (mature) gametophyte. Since the discovery of AGs in this species (Döpp, 1950), *P. aquilinum* commonly has been utilized as a source of AGs and a ‘positive control’ to test whether a given species is capable of responding to them (e.g. Yatskievych, 1993). Spores from up to four sporophytes per species were tested and each combination (paired with conspecific or *P. aquilinum*) was replicated three times. As a control, spores of each species also were sown without any mature gametophytes present. All plates were checked under a stereoscope for the presence of gametangia (Fig. 1) once a week for 12 wk. A species was considered as responding to either *P. aquilinum* or conspecific AGs if (1) archegonia were formed only in the absence of influencing gametophytes and (2) antheridia were formed in the presence of influencing gametophytes.

### Antheridiogen response meta‐analysis

In order to provide a broader sampling of taxa, we combined the data from our experiments with the results of previous studies (88 papers; Table S2) to create a dataset of all known AG production and responsiveness across ferns (Table S3). For each tested (target) species, we scored the following: the types of response tested (‘antheridium formation’ or ‘dark germination’), the outcome of the response (‘positive’ or ‘negative’; implicit evidence sometimes was considered), and the source species used to induce the response (e.g. the same species, a congener, or a control AG source such as *P. aquilinum*). Some species were tested with multiple source species. In cases where different responses were recorded from the same species combination, we recorded each as a separate data point. For some data points obtained from the literature, assays for responses were carried out not with exogenous AGs produced from gametophytes, but with gibberellins (which are known to produce an AG‐like effect in some responsive species; Näf *et al*., 1975). In these cases, 'gibberellin' was provided in place of the inducing species. In addition, we also scored ploidy level (diploid or polyploid) and reproductive mode (sexual or apomictic) for each species (Table S4). Ploidy data were obtained from floristic treatments, monographs and published chromosome counts; in some cases, data were not available. The reproductive mode of each taxon was obtained from Liu *et al*. (2012). Only species that are obligately apomictic were scored as apomictic.

The dataset enabled us to score each taxon as AG responsive or unresponsive (Table S4). Each species was considered as responsive to AG if at least one of these combinations yielded positive results. Five species were excluded from all additional analyses because they were either tested only against a type of AG inappropriate for the species (*Cibotium barometz* – gibberellin + *Pteridium aquilinum*, *Cyathea australis* – gibberellin + *P. aquilinum*, *Pentarrhizidium orientale* – gibberellin, *Radiovittaria stipitata* – *P. aquilinum*) or because conflicting data made it impossible to clearly label the species as responsive or not (*Phlebodium aureum*; Näf, 1956; Voeller, 1964; Weinberg & Voeller, 1969; Gemmrich, 1986; Chiou & Farrar, 1997). The first four species mentioned were removed as they are almost certainly false negatives and further testing is needed for a proper assessment of all five excluded taxa.

Additionally, our dataset allowed for re‐evaluating previously described AG groups/types. There are two conditions for each type to be unique. First, each taxon produces and/or reacts to only one AG type. If, for instance, one species reacted to two potential types, the types were merged into one. Second, taxa do not have to react to every single AG source within a type. As they represent a wide array of different chemicals, we do not consider the ‘gibberellin’ group to be AGs for the purposes of type delimitation.

In order to examine whether precocious antheridium formation and dark germination are tightly linked, we used our dataset to find any potential correlation. Species tested for both effects were evaluated as consistent (either affected under light and dark conditions or never) or inconsistent (affected only under one condition). Species tested for only one effect or against an inappropriate AG type (usually gibberellins failing to affect members of Polypodiales) were excluded from this comparison.

The correlation of AG production and response with species attributes (e.g. apomict/sexual, diploid/polyploid) was evaluated using chi‐square tests performed in R v.3.4.3 (R Core Team, 2017). Species whose ploidy level could not be determined were excluded from the diploid/polyploid comparison.

## Results

### Cultivation

A total of 68 species were cultivated and tested for AG response (Table 1). Of those, 56 species were tested with a conspecific AG source, and 25 reacted. Additionally, 44 species were tested using *P. aquilinum* as the source, and 22 reacted. Six species from the genera *Asplenium*, *Ctenitis*, *Cyathea* and *Pityrogramma* responded to conspecific but not to *P*. *aquilinum* AG. The opposite case, reacting only to *P. aquilinum* but not to conspecific AG, occurred for *Odontosoria* and *Phlebodium*.

**Table 1:** Overview of the response of 68 tested species to the cultivation experiment.

Tested species	Family	Response to self^1^	Response to *Pteridium aquilinum* ^1^
*Adiantum radicans*	Pteridaceae	Yes	Yes
*Asplenium adiantum‐nigrum*	Aspleniaceae	Not tested	Yes
*Asplenium auritum*	Aspleniaceae	Yes	No
*Asplenium cuneifolium*	Aspleniaceae	Not tested	Yes
*Asplenium ruta‐muraria*	Aspleniaceae	Not tested	No
*Asplenium scolopendrium*	Aspleniaceae	Not tested	No
*Asplenium septentrionale*	Aspleniaceae	Not tested	Yes
*Asplenium serratum*	Aspleniaceae	Yes	No
*Blechnum occidentale*	Blechnaceae	Yes	Yes
*Blechnum polypodioides*	Blechnaceae	Yes	Yes
*Bolbitis portoricensis*	Dryopteridaceae	No	No
*Campyloneurum aphanophlebium*	Polypodiaceae	No	No
*Campyloneurum brevifolium*	Polypodiaceae	Yes	Not tested
*Christella dentata*	Thelypteridaceae	Yes	Yes
*Cibotium menziesii*	Cibotiaceae	Yes	No
*Ctenitis sloanei*	Dryopteridaceae	No	Not tested
*Cyathea microdonta*	Cyatheaceae	Yes	No
*Cyathea multiflora*	Cyatheaceae	Yes	No
*Davallia fejeensis*	Davalliaceae	Yes	Not tested
*Diplazium striatastrum*	Athyriaceae	No	No
*Draconopteris draconoptera*	Tectariaceae	No	Not tested
*Dryopteris carthusiana*	Dryopteridaceae	Not tested	Yes
*Dryopteris caucasica*	Dryopteridaceae	Not tested	Yes
*Dryopteris dilatata*	Dryopteridaceae	Not tested	No
*Dryopteris expansa*	Dryopteridaceae	Not tested	Yes
*Dryopteris filix‐mas*	Dryopteridaceae	Not tested	Yes
*Dryopteris oreades*	Dryopteridaceae	Not tested	Yes
*Elaphoglossum latifolium*	Dryopteridaceae	Yes	Not tested
*Elaphoglossum peltatum*	Dryopteridaceae	No	No
*Equisetum arvense*	Equisetaceae	No	No
*Equisetum fluviatile*	Equisetaceae	No	No
*Equisetum palustre*	Equisetaceae	No	Not tested
*Equisetum sylvaticum*	Equisetaceae	No	Not tested
*Goniopteris curta*	Thelypteridaceae	No	Not tested
*Goniopteris nicaraguensis*	Thelypteridaceae	Yes	Yes
*Hypoderris brauniana*	Tectariaceae	Yes	Yes
*Lomariopsis japurensis*	Lomariopsidaceae	No	Not tested
*Lomariopsis vestita*	Lomariopsidaceae	No	No
*Lygodium japonicum*	Lygodiaceae	Yes	Not tested
*Lygodium microphyllum*	Lygodiacea	Yes	Not tested
*Macrothelypteris torresiana*	Thelypteridaceae	No	No
*Meniscium lingulatum*	Thelypteridaceae	Yes	Yes
*Mickelia nicotianifolia*	Dryopteridaceae	No	Not tested
*Microgramma lycopodioides*	Polypodiaceae	No	Not tested
*Zealandia pustulata*	Polypodiaceae	No	No
*Nephrolepis biserrata*	Nephrolepidaceae	No	No
*Odontosoria c.f. gymnogrammoides*	Lindsaeaceae	No	Yes
*Oleandra articulata*	Oleandraceae	Yes	Not tested
*Olfersia cervina*	Dryopteridaceae	No	No
*Osmunda claytoniana*	Osmundaceae	No	Not tested
*Osmunda regalis*	Osmundaceae	No	Not tested
*Osmundastrum cinnamomeum*	Osmundaceae	No	Not tested
*Parapolystichum excultum*	Dryopteridaceae	Yes	Not tested
*Pecluma pectinata*	Polypodiaceae	No	Not tested
*Phlebodium pseudoaureum*	Polypodiaceae	No	Yes
*Pityrogramma calomelanos*	Pteridaceae	Yes	No
*Pleopeltis furfuracea*	Polypodiaceae	No	Not tested
*Polybotrya osmundacea*	Dryopteridaceae	No	Not tested
*Polystichum munitum*	Dryopteridaceae	No	No
*Polystichum setiferum*	Dryopteridaceae	Not tested	Yes
*Pteris propinqua*	Pteridaceae	Yes	Yes
*Saccoloma elegans*	Saccolomataceae	Yes	Yes
*Saccoloma inaequale*	Saccolomataceae	Yes	Not tested
*Salpichlaena volubilis*	Blechnaceae	No	No
*Serpocaulon triseriale*	Polypodiaceae	Yes	Yes
*Tectaria heracleifolia*	Tectariaceae	No	Not tested
*Thelypteris kunthii*	Thelypteridaceae	Yes	Yes
*Trichomanes diversifrons*	Hymenophyllaceae	No	Not tested

^1^ Taxa that responded formed antheridia first when exposed to an archegoniate conspecific or *Pteridium aquilinum* gametophyte, despite forming archegonia first under control conditions.

### AG meta‐analysis

#### Datasets

**Fig. 2:**
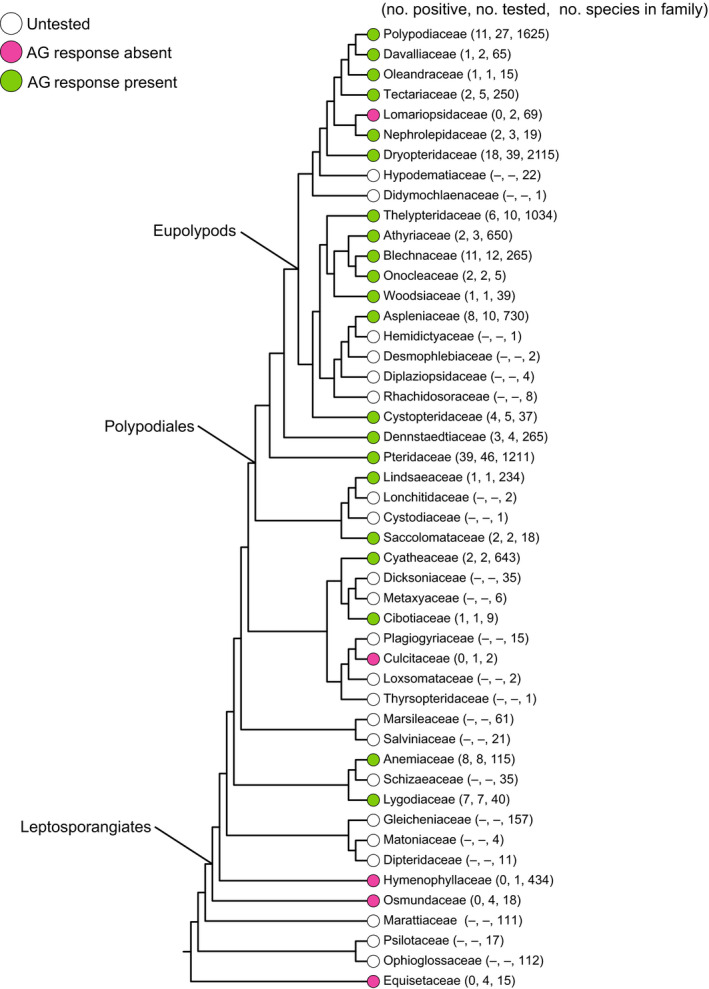
Fern phylogeny (with relationships based on PPG 1, 2016) indicating the families tested for antheridiogen (AG) response. The number of responsive, tested and total species in a family also is given.

**Fig. 3:**
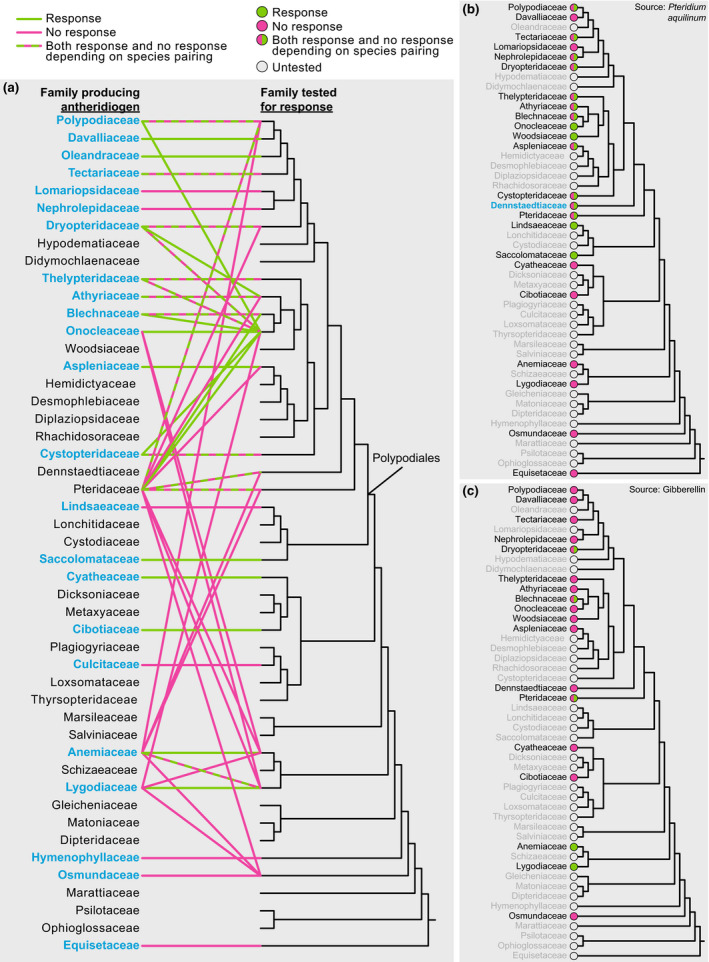
Overview of antheridiogen interactions (response or no response) between tested fern taxa on a family level (phylogeny tree based on PPG 1, 2016): (a) interactions between all families, excluding *Pteridium aquilinum* (Dennstaedtiaceae) as antheridiogen producer (families tested labelled blue); (b) response of taxa to *P. aquilinum* (Dennstaedtiaceae, labelled blue); and (c) response to gibberellins.

**Fig. 4:**
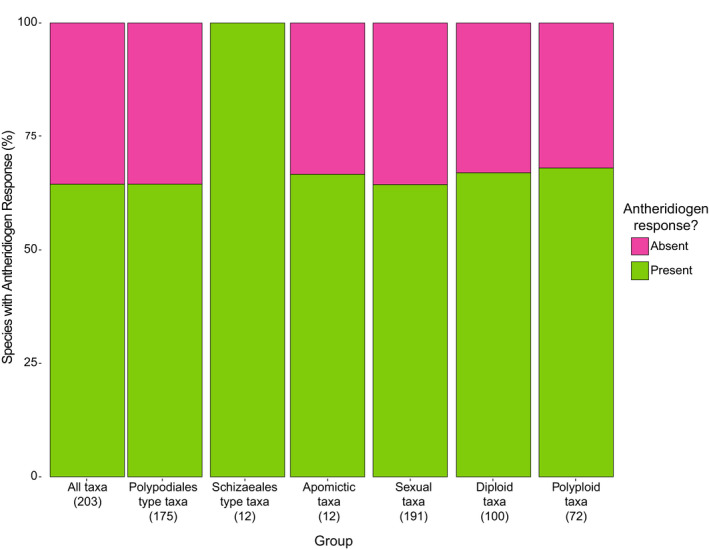
Overview of the percentage of fern taxa reacting to antheridiogens. The percentage of all studied taxa is listed alongside those for various subgroups based on antheridiogen type (Polypodiales‐type AG (AGPo)/ Schizaeales‐type AG (AGSc); others not listed), ploidy level (diploid/polyploid) and reproductive types (apomictic/sexually reproducing).

The final dataset (Table S3) included a total of 208 species from 26 families, involved in a total of 498 pairings, either with a conspecific or another taxon (Figs 2, 3). After the exclusion of five species (see the Materials and Methods section), 64.5% of the 203 taxa responded to AGs (Fig. 4). Interestingly, three species (*Cyrtomium fortunei*, *C. macrophyllum* and *Polystichum lonchitis*) seemed to produce AG but did not react to any tested AG source. Tested representatives of five families (Culcitaceae, Equisetaceae, Hymenophyllaceae, Lomariopsidaceae and Osmundaceae) did not appear to produce or respond to AG at all (Fig. 2).

#### Antheridiogen types

From our dataset, we identified two main AG types, corresponding to the *Pteridium* and *Anemia* types affecting Polypodiales and Schizaeales, respectively (Fig. 3a). In total, 64.6% of the tested representatives of Polypodiales responded to AG (usually from *P. aquilinum*; Fig. 3b) and response to gibberellin was extremely rare (Fig. 3c). All representatives of Schizaeales responded to some form of AG (Fig. 4) and to supplemented gibberellins (Fig. 3c), if tested. Using our definition (in Methods), we also identified several different potential minor AG types, affecting only a single species or genus. Based on our definition, many species were considered as having their own type only because they have not been tested against any other species (*Davallia fejeensis*, *Elaphoglossum latifolium*, *Gymnocarpium disjunctum*, *Oleandra articulata*, *Parapolystichum excultum*, *Polypodium cambricum*, *Sadleria* spp., *Woodwardia radicans*) or were cross‐tested within a small group of species (*Cheilanthes* spp.). These taxa all belong to the order Polypodiales. *Thelypteris ovata* and *Hemionitis palmata* only failed to respond to gibberellins and *Pteris vittata*, respectively, but their congener responded to *P. aquilinum*. Three species of *Asplenium* failed to react to *P. aquilinum* (*Asplenium auritum*, *Asplenium serratum*) and gibberellins (*Asplenium ruta‐muraria*), but they are not phylogenetically closely related and other *Asplenium* species (*A. cuneifolium*, *A. septentrionale*) respond to *Pteridium*‐type AG. *Pityrogramma calomelanos* successfully influenced itself and two species of *Onychium* but failed to react to *P. aquilinum*. Related *Pityrogramma* species also did not respond to *Pteris vittata* AG. These results indicate that a distinct AG system may be operating within *Pityrogramma*. Likewise, V*ittaria* spp. gemmae responded to exudates of long‐lived congeneric gametophytes and supplemented gibberellins by forming antheridia. *Pteridium aquilinum* AG failed to induce the same response. This would indicate an AG system similar to the Anemia type. *Ceratopteris* spp. were affected only by conspecific AG but failed to respond to *Anemia* spp.*, P. aquilinum* and *Pteris vittata*. *Ceratopteris* AG also failed to influence *Bommeria* species responsive to *P. aquilinum*. It is uncertain whether *Ceratopteris* AG would be needed in higher concentrations for any effect to occur, or perhaps it is distinct or different enough to be unable to affect the few species tested but still within the *Pteridium* type. Outside of Polypodiales, three tree fern (Cyatheales) species (*Cibotium menziesii*, *Cyathea microdonta*, *Cyathea multiflora*) responded to conspecific AG but not to *P. aquilinum*. Related species (*Cibotium barometz*, *Cyathea australis*) also failed to respond to gibberellins, indicating that tree ferns may utilize chemically and phylogenetically different AGs belonging to one or multiple types.

#### Dark germination

Data on dark germination were obtained for 53 taxa. Data were sufficient (see Methods) to evaluate 32 taxa (20.4% of all taxa with determined AG response). In this subset, 26 taxa (81.3%) germinated in darkness. In three cases, the dark germination response was different than the observed antheridium induction response: *Ceratopteris thalictroides* and *Thelypteris ovata* did not germinate in darkness despite being influenced under light, and *Polypodium cambricum* germinated in darkness despite not responding to AG under light.

#### Reproductive types and polyploidy

Overall, 12 (6%) of the taxa sampled were obligately apomictic. Apomictic and sexual taxa had similar (i.e. not significantly different) response rates to AGs of 66.7% and 64.4%, respectively (χ^2^ < 10^−6^; df = 1; *P* = 1; Fig. 4). Of the 208 taxa included in the dataset, ploidy level and AG response were determined for 172 taxa. The 100 diploid and 72 polyploid taxa had nearly equal response rates of 67.0% and 68.1%, respectively (χ^2 ^= 0; df = 1; *P* = 1; Fig. 4).

## Discussion

### Antheridiogen data synthesis and meta‐analysis

Combining our results with published data from the literature, we present an updated list of 208 fern species from 26 families that have been tested for antheridiogen (AG) response or production (Table S4). Unlike previous major reviews on the topic (Näf et al., 1975; Raghavan, 1989; Schneller, 2008), we have recorded the response of each species to all tested AG sources (Table S3). A recent estimate puts the number of fern species at 10 578 (PPG 1, 2016), meaning that 2% of all known fern species have now been tested for AG activity. This is a substantial increase from the < 1% tested that was estimated by Kirkpatrick & Soltis (1992). However, the vast majority of fern diversity remains unstudied. Athyriaceae and Cyatheaceae deserve special attention as these are species‐rich families with only three species tested each.

About two‐thirds (64.5%) of all tested species responded positively to some type of AG. Additionally, three species produced AG but did not react to the pheromone. Clearly, AGs play a major role in the lives of fern gametophytes. Nevertheless, the real percentage of fern species responding may be different. Responsive species may be over‐represented in our dataset, as negative results are less likely to be published. However, some species may be incorrectly labelled as nonresponsive if they failed to respond to some of the model AG sources. For example, *Pentarrhizidium orientale* failed to react to gibberellins (Weinberg & Voeller, 1969). Labelling the species as nonresponsive based only on this result could be misleading (the species was excluded as a false negative) as the closely related *Onoclea sensibilis* reacts to AG of ≥ 27 other species, but not to gibberellins. Not all cases can be as clear as that of *Pentarrhizidium orientale* and AG systems are too complex to accurately assign species as nonresponsive based on a limited number of pairings.

### Antheridiogen types

Our dataset clearly demonstrates two major AG types, one affecting the order Polypodiales and the other affecting Schizaeales. Among the many minor types described, most would likely fall within the main Polypodiales type, if more pairings are conducted. Some of these minor types (e.g. *Asplenium, Pityrogramma, Vittaria*) are supported with inconclusive evidence and require further study. *Ceratopteris* is generally considered to have its own AG type (Schneller *et al*., 1990) and this is supported by the most convincing evidence, such as the lack of response to multiple other species and no germination in darkness. However, we would like to caution against an unambiguous distinction of the *Ceratopteris* type until more pairings are done with other Polypodiales species, especially from Pteridaceae. Some sort of AG system also operates in tree ferns (Cyatheales) that seems distinct from the two major AG types. However, we have insufficient data on the chemical nature and the extent of influence of this system. Tree ferns clearly deserve more study.

In accordance with our merger of many minor types to the larger ones, we would like to suggest the following naming convention: The types should be named after the broadest group under their influence; for example, Polypodiales‐type AG, Schizaeales‐type AG, Cyatheales‐type AG (for a possible unified tree fern AG type) and Ceratopteris‐type AG (for the potential *Ceratopteris* type). To allow for easier reading and understanding we advise abbreviating the types as AGPo, AGSc, AGCy and AGCe, respectively, avoiding the usually applied subscript.

### Evolution of antheridiogens

Pheromonal control of sex expression via AGs is widespread among leptosporangiate ferns and has likely evolved multiple times. To understand the evolution of AGs, phylogeny must be considered. For the purpose of this analysis, we presume that only three main types (one being a unified Cyatheales type) described above are distinct. All pairings involving *Equisetum* indicate that it has no AG system, suggesting that AGs evolved within the ferns after the divergence of *Equisetum*. No other eusporangiate ferns have yet been tested, but studies of sexual expression in Osmundaceae, which are sister to all other leptosporangiate ferns, indicate a potential pheromonal control different from AG (Hollingsworth *et al*., 2012). Phylogenetically, the three types of AG system we recognize could represent three separate origins. The first true AG system is that found in the order Schizaeales, chemically based on gibberellins (Fig. 3c). The origin of this AG type is uncertain until denser sampling of non‐polypod leptosporangiates is achieved. Nevertheless, it could be either that the order represents an independent acquisition of AGs, or that AGs were present in the common ancestor of the entire group (Schizaeales through Polypodiales), and the system was later lost in water ferns and its chemical nature was changed considerably in other groups. Therefore, it seems more likely that the Schizaeales type system evolved independently within that order. The second origin, or perhaps multiple origins, appeared in Cyatheales. However, our knowledge in this group is scarce and further research is required. The third origin of AG, the Polypodiales type, could have evolved right at the origin of Polypodiales, potentially as a key innovation of this highly diverse lineage. Our results indicate the presence of AG activity in *Saccoloma* and Lindsaeaceae (Fig. 3a,b). Antheridiogens are certainly well‐established within Pteridaceae, as four of its five subfamilies, including the Cryptogrammoideae, which are sister to the other four (Schuettpelz *et al*., 2007), have species responsive to AG, and members of other families in Polypodiales react to Pteridaceae AG (Fig. 3a). Further studies of *Saccoloma*, *Cystodium*, *Lonchitis* and Lindsaeaceae will be critical for establishing the origins of AGs within Polypodiales, and studies of non‐leptosporangiates are necessary to understand the evolution of antheridiogens across all ferns.

Although we advocate for a broader AG type concept, it is important to note that AGs likely diversify considerably within each type. A considerable number of various distinct chemical entities were described within Schizaeales (Nakanishi *et al*., 1971; Endo *et al*., 1972; Nester *et al*., 1987; Yamane *et al*., 1988; Yamauchi *et al*., 1996; Wynne *et al*., 1998). In this order, all species reacted positively to congeners, if tested. However, the compatibility between the two families Anemiaceae and Lygodiaceae was limited. No chemical compound was fully described within Polypodiales, but the lack of compatibility across some families is reminiscent of what is seen in Schizaeales. For example, members of Pteridaceae are capable of inducing a response in members of Onocleaceae and Blechnaceae, but not Aspleniaceae (Fig. 3a). This phenomenon could be caused by the lack of selective pressure to conserve the chemical structure of AG. The chemical compounds may diversify in each lineage, being less capable of affecting evolutionarily distant species. A possible result would be the evolution of new types, for example in *Ceratopteris*.

*Ceratopteris* is of particular interest when considering the evolution of ferns in association with AG systems. This genus is the only representative of homosporous aquatic ferns. Its species also form the largest spores of all homosporous ferns (Tryon & Lugardon, 1991) and it may have its own unique AG type. In theory, plants in aquatic environments benefit from propagules with higher energy reserves to speed up the life cycle and help survive in a carbon‐dioxide‐poor environment (Petersen & Burd, 2017). A greater abundance of male gametes also is beneficial to increase the chance of mating in water. Heterospory, in which a few large megaspores and many small microspores are produced, fits perfectly into this environment. Unsurprisingly, the known cases of heterospory in ferns involve the true water ferns (Salviniales) and *Pteris platyzomoides*, a unique species growing in seasonally waterlogged habitats (Tryon, 1964). *Ceratopteris* represents an alternative solution to this problem. Large spores provide the energy reserves, and the AG system guarantees an overabundance of male gametes in populations whereas single spores can still grow into bisexuals and colonize new habitats. In *Ceratopteris*, the AG system does not just substitute the genetic variation aspect of heterospory, but also confers the benefits of heterospory in aquatic environments. It is possible that the ancestors of Salviniales developed heterospory, in part, due to a lack of AG system in their lineage. In turn, *Ceratopteris* might have become fully heterosporous were it not for AGs.

### Germination of spores in darkness

In addition to stimulating precocious formation of antheridia, AGs also enable germination of spores in darkness. Of the 32 species evaluated, 80% reacted to AG, compared to the 64% overall reaction. This higher response rate is likely caused by the over‐representation of Schizaeales (which all react) in the dark germination subset. Furthermore, 29 of 32 species (90.6%) were consistent in their response. The first exception was *Polypodium cambricum* germinating in darkness, but without induced antheridia in illuminated cultures (Welling & Haufler, 1993). However, some members of Polypodiaceae are known to respond only to high concentrations of AG (Chiou & Farrar, 1997) and it is possible that the concentration of AG used by Welling & Haufler (1993) was insufficient to promote antheridium formation in illuminated cultures. The second exception, *Thelypteris ovata*, failed to germinate in darkness despite responding to AG in light (Nester‐Hudson *et al*., 1997). However, germination percentages were checked only 7 d after sowing, a duration equal to the time needed for germination in light for the species. As dark germination may take longer than germination under normally illuminated conditions (Weinberg & Voeller, 1969), it is possible that AG‐induced dark germination would have been observed at a later point. Finally, *Ceratopteris thalictroides* does not induce germination in darkness (Schedlbauer, 1976). Spores of *Ceratopteris* generally do not germinate in darkness, although some exceptions have been reported (Scott & Hickok, 1987). From our data, this is the only clear example of AG being capable of inducing antheridiogenesis but not germination in darkness. With the notable exception in *Ceratopteris*, germination in darkness seems to be a reliable indicator of antheridiogen response in fern species. If done properly, assays of dark germination could be used as this method is less demanding than its light counterpart and lends itself to mass analysis of the many species yet to be studied.

### Antheridiogens in apomictic ferns

Apomictic ferns, which produce sporophytes spontaneously from gametophytes without fertilization (Grusz, 2016), can produce and respond to AG. Usage of AG in apomicts presents an interesting evolutionary dilemma. On the one hand, apomictic gametophytes that respond to AGs and produce antheridia may be wasting valuable resources to do so, and any subsequent slowed growth might limit their ability to form sporophytes apomictically. On the other, an appealing possibility is that production of AGs may confer a competitive advantage to apomictic fern gametophytes over co‐occurring sexual taxa, as some apomictic gametophytes grow faster than their sexual competitors (Whittier, 1968; Haufler & Gastony, 1978). Theoretically, a disturbance revealing a new niche for ferns could be colonized by fast‐growing apomictic gametophytes that would suppress sexual gametophytes of similar age and any latecomers. Response to AG in apomicts would then be irrelevant, as older gametophytes producing AG themselves are insensitive to it (Näf, 1958). Provoking dark germination and subsequent antheridium formation in subterranean spores would have the added effect of depleting the spore bank, thus limiting potential future competition.

Like sexual species, about two thirds of apomictic species respond to AG. To date, assessment of AG responsiveness in apomict‐containing lineages is too limited to draw broad conclusions, and evaluation of their responses needs to be tested in a phylogenetic context. However, we present several possible explanations for the similar response between apomictic and sexually reproducing taxa. First, apomicts arise from sexual ancestors (Grusz, 2016) and AG response is therefore inherited from them, and thus may be evolutionarily conserved within some lineages. In *Cheilanthes* and *Cystopteris*, the AG‐responsiveness of an allotetraploid species was reported as the average of its diploid progenitors, suggesting a legacy of AG activity in descendants (Haufler & Ranker, 1985; Pajarón *et al*., 2015). Many apomicts start as hybrids (Grusz *et al*., 2009; Liu *et al*., 2012; Ekrt & Koutecký, 2016) and likely follow a similar pattern. Yatskievych (1993) found that two apomictic *Cyrtomium* species retained the ability to produce AG but did not react to it, thus keeping the advantage, but losing the liability. The simple inheritance of AG systems from ancestors may be the most parsimonious explanation of the equal usage of AGs among sexual and apomictic taxa. Nevertheless, the presence of apomictic species that have lost sensitivity to AG despite being able to synthesize it indicates that adaptive pressures affect the use of the AG system in apomicts.

Second, apomicts may adapt not by losing the ability to respond to AGs, but instead by increasing the needed concentration of the pheromone. Once the species is insensitive enough that common competitors and their typical AG output cannot influence it, there would be no selective pressures to reduce sensitivity further. Schneller (1981) reported AG effects to be weaker in apomictic members of the *Dryopteris affinis* group compared to sexually reproducing *D. filix‐mas*. In the same genus, sexually reproducing *D. carthusiana* reacts to AG of *Pteridium aquilinum*, but not to congeneric species that it competes with (Testo *et al*., 2015). Thus, in laboratory experiments, unusually high concentrations or slightly different sources of AG may result in a positive response in otherwise insensitive species.

Finally, response to AG may be adaptive for apomicts. As mentioned above, apomicts may use AGs to suppress sexual competitors. Furthermore, related apomictic and sexually reproducing ferns may hybridize to form semi‐fertile hybrids, which then reproduce via apomixis (Ekrt & Koutecký, 2016). This peculiar merger likely happens via fertilization of an egg from a sexual species by an apomict's sperm, as most apomicts do not form archegonia (Döpp, 1959; Whittier, 1968; Yatskievych, 1993, but see Hori & Murakami, 2019). This way, sporophyte‐bearing apomictic gametophytes not only reduce future competition by suppressing conspecific gametophytes, but the suppressed apomictic gametophytes also flood sexual gametophytes that made it to the archegoniate phase with interspecific sperm. Likewise, a sexual archegoniate gametophyte growing on top of an apomictic spore bank may be forced to hybridize this way. In both cases, sexually reproducing gametophytes are either denied fully functioning sporophytic offspring or end up propagating the genes of the apomict. However, owing to the lack of testing in field conditions, we cannot be sure how important this competition between apomicts and sexually reproducing species really is.

### Antheridiogens and polyploidy

Polyploidy plays an important role in fern evolution (Vida, 1976; Wood *et al*., 2009; Liu *et al*., 2019). Most ferns use a mixed‐mating system (Haufler *et al*., 2016), forming sporophytes by self‐fertilization (gametophytic selfing) or by exchanging gametes with other gametophytes (sporophytic selfing or outcrossing). The use of AG promotes the latter option as unisexual gametophytes are more likely to occur. However, polyploid ferns should in theory be more tolerant of forming progeny by self‐fertilization on bisexual gametophytes (Masuyama, 1979; Soltis & Soltis, 2000; Pangua *et al*., 2003; Flinn, 2006; Testo *et al*., 2015; Sessa *et al*., 2016). As sensitivity towards AGs limits the ability to form bisexual gametophytes, polyploids might be more likely to abandon the use of AGs. That way, each polyploid gametophyte can self‐fertilize and take advantage of their inherent genetic diversity (Hickok, 1978). However, the ratio of responsive diploids and polyploids is nearly identical (Fig. 4). As in apomicts, the optimal strategy for a predominantly selfing species may be to exude AGs but not react to them. This strategy has been described in the tetraploid *D. carthusiana* (Testo *et al*., 2015). As mentioned above, removing the inherited sensitivity towards AG may be a long and difficult process, but polyploids are often evolutionarily young. Alternatively, Schneller & Hess (1995) suggest that AGs in tetraploid *Asplenium ruta‐muraria* may be used to increase the quantity of available sperm in their environments, where water is a factor limiting fertilization (such as in the rock walls that *A. ruta‐muraria* typically inhabits). The gametophytic community in such a habitat might be founded by a single sporophyte, so the end goal is not increased genetic diversity but an increased chance of fertilization. Finally, polyploids may still benefit from the outcrossing supported by AGs and the positives of retaining their AG sensitivity outweigh the potential negatives of selfing with little genetic risk.

### Conclusion

This comprehensive meta‐analysis of 88 published papers together with new data from cultivation experiments has focused on the occurrence of antheridiogens in ferns, especially from the perspective of phylogeny, dark germination, mating modes and ploidies. The meta‐analysis shows that the AG system is widespread among ferns. About two‐thirds (64.5%) of all tested species responded positively to AGs. This finding demonstrates their far‐reaching importance, likely related to consequences that affect many aspects of fern reproduction. This unique system of sex determination and ensuing population demographic control deserves more interest. Seventy years after the discovery of AGs by Walter Döpp (Döpp, 1950) the vast majority of fern species (98%) remains unexplored. We suggest that large, species‐rich families such as Athyriaceae and Cyatheaceae, with barely any species tested, should be the subject of future inquiries, as should the non‐leptosporangiate fern lineages. Several AG types are well‐established by now, but others still require thorough testing to determine their scope, distinctness, and features.

Despite expectations, the majority (66.7%) of apomictic species surveyed to date respond to AG. The consequences of this may play an important role in survival and competition among fern gametophytes in nature as well as interactions between apomictic and sexually reproducing taxa. Our study also suggests that there is no difference between diploids (67.0%) and polyploids (68.1%) in response to AGs, so the pheromonal system may be advantageous even to species capable of being predominantly selfing. Finally, there is a strong correlation between germination in darkness and precocious antheridium formation in light. Testing for dark germination can be done through more expedient methods with binary results. These methods may be key to mass testing AG response in many of the yet unstudied species. We are now beginning to understand how AGs operate on the molecular level (Valledor *et al*., 2014; Ganger *et al*., 2015; Attalah *et al*., 2018; Chen *et al*., 2019) but many questions about their distribution and evolution remain unanswered. Hopefully, our comprehensive dataset can provide a starting point for fern researchers to learn whether their species of interest use this intriguing system of pheromonal control over sexual determination.

## Author contributions

OH, WLT, JEW and LE designed the study; OH, WLT, CEC and JP conducted the cultivation experiments; OH compiled the meta‐analysis list; and OH, WLT and EBS analyzed the data. All authors contributed to the writing of the manuscript.

## Supporting information


**Tables S1** List of taxa cultivated in this study.
**Table S2** List of literature used to compile interactions dataset.
**Table S3** List of antheridiogen interactions between fern taxa (meta‐analysis + cultivation results).
**Table S4** List of all taxa (meta‐analysis + cultivation results) determined as responsive or not to antheridiogens with additional information for each taxon.
Please note: Wiley Blackwell are not responsible for the content or functionality of any Supporting Information supplied by the authors. Any queries (other than missing material) should be directed to the *New Phytologist* Central Office.
